# Pirt, a TRPV1 Modulator, Is Required for Histamine-Dependent and -Independent Itch

**DOI:** 10.1371/journal.pone.0020559

**Published:** 2011-05-31

**Authors:** Kush N. Patel, Qin Liu, Sonya Meeker, Bradley J. Undem, Xinzhong Dong

**Affiliations:** 1 The Solomon H. Snyder Department of Neuroscience, Center for Sensory Biology, Johns Hopkins University School of Medicine, Baltimore, Maryland, United States of America; 2 Department of Medicine, Johns Hopkins University School of Medicine, Baltimore, Maryland, United States of America; 3 Howard Hughes Medical Institute, Chevy Chase, Maryland, United States of America; Sackler Medical School, Tel Aviv University, Israel

## Abstract

Itch, or pruritus, is an important clinical problem whose molecular basis has yet to be understood. Recent work has begun to identify genes that contribute to detecting itch at the molecular level. Here we show that Pirt, known to play a vital part in sensing pain through modulation of the transient receptor potential vanilloid 1 (TRPV1) channel, is also necessary for proper itch sensation. *Pirt^−/−^* mice exhibit deficits in cellular and behavioral responses to various itch-inducing compounds, or pruritogens. Pirt contributes to both histaminergic and nonhistaminergic itch and, crucially, is involved in forms of itch that are both TRPV1-dependent and -independent. Our findings demonstrate that the function of Pirt extends beyond nociception via TRPV1 regulation to its role as a critical component in several itch signaling pathways.

## Introduction

The familiar sensation of itch has emerged as an important topic of research. Despite its great clinical significance, little is known about itch, especially in comparison to other sensory modalities [1]. In particular, efforts to investigate the molecular and cellular bases of itch sensation [2] are gaining momentum as pruritoception is still poorly characterized at these levels.

Previous work from our laboratory identified a novel gene encoding a membrane protein specifically expressed in sensory neurons [3]. *Pirt*, which is highly conserved among vertebrates, contributes to thermal nociception dependent upon the capsaicin receptor TRPV1 and regulates its physiology accordingly [3]–[5]. Pirt binds to both phosphatidylinositol (4,5)-bisphosphate (PIP_2_) and TRPV1 and is necessary for PIP_2_-dependent activation of TRPV1 current. The sensory-specific expression pattern of Pirt and its ability to bind TRPV1 and other ion channels as well as PIP_2_ suggest additional modulatory functions and one promising candidate is pruritoception.

TRPV1 has previously been implicated in itch induced by histamine, the most commonly studied pruritogen. *Trpv1^−/−^* mice show a scratching deficit in response to histamine injection [6], [7] and the presence of TRPV1 is required for proper activation of the histamine H1 receptor [6]. At a cellular level, TRPV1^+^ neurons play a major role in itch mediated by several pruritogens including, but not limited to, histamine [7], [8]. How TRPV1 fits into the broader picture of histamine-dependent and -independent itch is uncertain. The possible role of Pirt in these processes, which we have identified as a critical modulator of TRPV1 function, has yet to be investigated.

Moreover, we have shown Pirt can bind other TRPs [3] and it may regulate other ion channels as well. Expression of Pirt is widespread among dorsal root ganglion (DRG) neurons, spanning the entire small- and medium-diameter population. Its functional interaction with PIP_2_ also hints at other roles in the physiology of sensory neurons. Working from the hypothesis that Pirt modulates TRPV1 and potentially other itch-associated proteins, we used the Pirt null mouse in conjunction with behavioral and imaging methods to examine the function of Pirt in the context of itch sensation.

## Results

### Pirt is essential for proper detection of histaminergic itch

**Figure 1:**
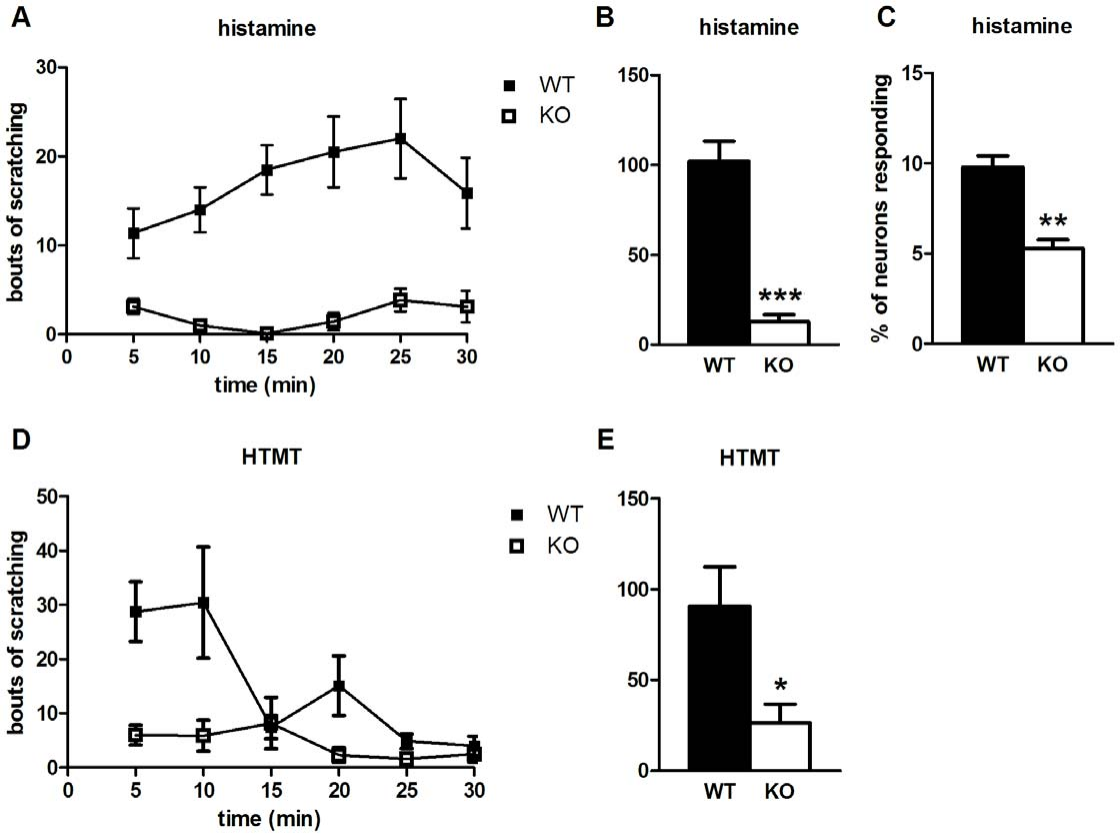
Pirt contributes to histaminergic itch. (**A**) The time course shows scratching bouts, presented as mean ± SEM, in response to histamine injection (10 µmol) over a 30 min period divided into 5 min intervals. (**B**) Total bouts of scratching over the 30 min observation period show *Pirt^−/−^* mice (n = 7) have decreased behavioral responses to histamine compared to WT mice (n = 8). (**C**) Ca^2+^ imaging shows fewer DRG neurons from Pirt KO mice respond to histamine (50 µM) than WT mice (n = 5 per genotype). (**D**, **E**) Pirt mutant mice (n = 10) also scratch less upon injection of the histamine H1 receptor-selective agonist HTMT (0.1 µmol) than WT (n = 9). * *p*<0.05, ** *p*<0.001, *** *p*<0.0001; two-tailed unpaired *t*-test.

We used a well-established assay to measure behavioral responses of mice to various itch-inducing compounds [9]. Histamine is known to generate a form of itch that is TRPV1-dependent [6], [7], suggesting a role for Pirt in this process. As predicted, histamine produces itch in wild type mice (108 bouts of scratching) that is severely reduced in *Pirt^−/−^* littermates (12 bouts, Fig. 1A, B). In addition to producing a behavioral scratch response, many pruritogens can directly activate DRG neurons *in vitro* to generate intracellular calcium and/or electrophysiological responses. Histamine has been shown to directly activate DRG neurons and this response is decreased in the presence of a TRPV1 antagonist [6], [10]. The percentage of neurons responding to histamine is significantly reduced in DRG cultures from *Pirt^−/−^* mice (5.3%) compared to wild type controls (9.8%, Fig. 1C). Histamine trifluoromethyl toluidide (HTMT), an agonist selective for the histamine H1 receptor [11], can also induce scratching, and this is strongly attenuated in *Pirt^−/−^* mice (91 bouts in WT vs. 27 bouts in Pirt KO, Fig. 1D, E). These data suggest a contribution of Pirt to histaminergic itch signaling through the H1 receptor, although another related protein, the histamine H4 receptor, may also be involved in histamine-dependent itch [12], [13].

### Pirt plays a crucial role in nonhistaminergic forms of itch

While histamine is a classical itch mediator that has proven fruitful for studying pruritoception, it is now clear nonhistaminergic types of itch exist that signal through separate pathways [1]. This is of great clinical significance as many pathological forms of itch are refractory to antihistamine treatment [14]. We next investigated the involvement of Pirt in nonhistaminergic itch.

**Figure 2:**
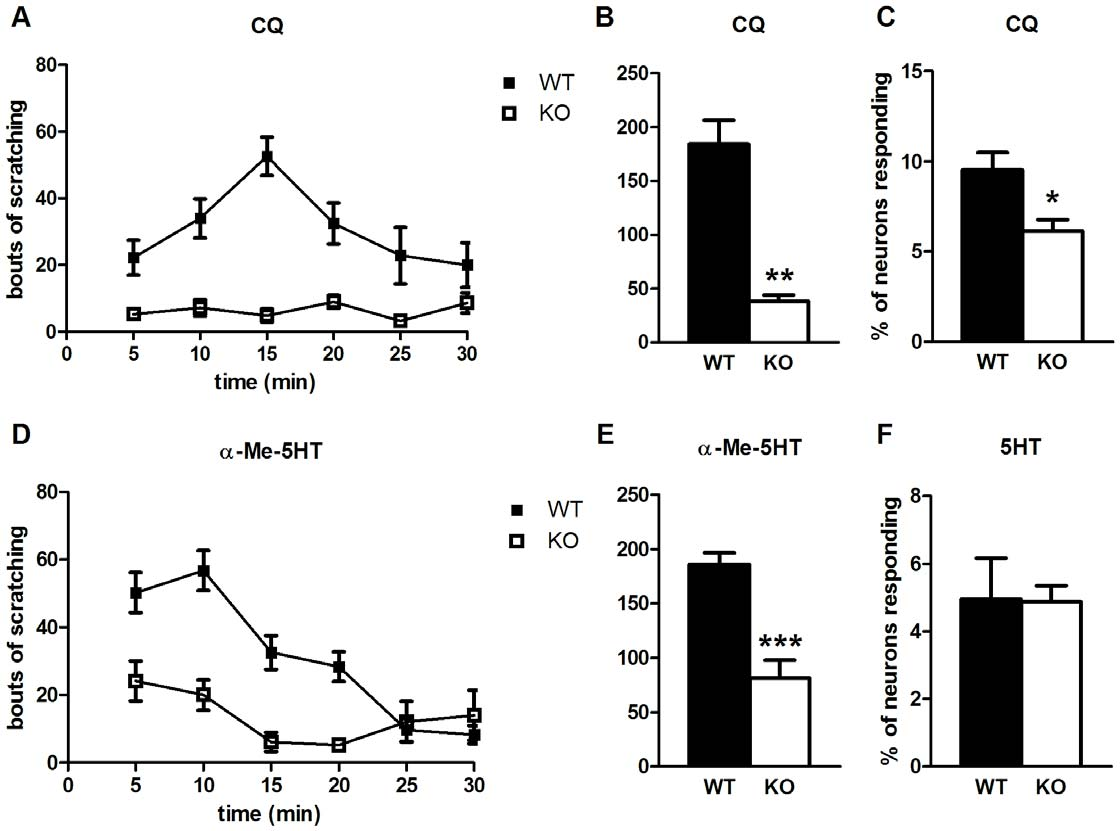
Nonhistaminergic itch requires Pirt. (**A**, **B**) CQ (200 µg) induces significantly less scratching behavior in *Pirt^−/−^* mice (n = 9) than WT controls (n = 8). (**C**) A smaller percentage of Pirt KO neurons respond to CQ (1 mM) compared to WT (n = 5 per genotype). (**D**, **E**) The 5HT_2_-selective agonist α-Me-5HT (30 µg) produces decreased scratching in KO mice (n = 8; WT, n = 13). (**F**) Responses to 5HT (10 µM) are unchanged in Pirt KO DRG neurons (n = 4 per genotype). * *p*<0.05, ** *p*<0.001, *** *p*<0.0001; two-tailed unpaired *t*-test.

The antimalarial drug chloroquine (CQ) commonly produces itch as a side effect [15], [16] and this cannot be ameliorated by antihistamines [17], [18]. CQ also induces itch when injected into mice and is transduced in a histamine-independent manner by the G protein-coupled receptor (GPCR) MrgprA3 [19]. Pirt mutant mice show a severe reduction in the behavioral itch response to CQ (38 bouts in KO vs. 184 in WT, Fig. 2A, B). Accordingly, fewer DRG neurons from *Pirt^−/−^* mice (6.1%) respond to CQ compared to WT neurons (9.5%, Fig. 2C).

Serotonin (5HT) is a well-known neurotransmitter that can also produce itch [20], [21]. We tested the response of *Pirt^−/−^* mice to alpha-methyl-serotonin (α-Me-5HT), a 5HT derivative that acts as a selective agonist for the 5HT_2_ family of receptors and is able to induce a scratching response [7], [22]. WT mice scratch more than twice as often (186 bouts) as Pirt mutants (82 bouts, Fig. 2D, E). Interestingly, DRG neurons in culture do not exhibit Ca^2+^ responses to α-Me-5HT, while responses to 5HT itself are unchanged in neurons from *Pirt^−/−^* mice (5.0% for WT against 4.9% in KO, Fig. 2F). This suggests an indirect mechanism of itch generated by α-Me-5HT, e.g. activation of 5HT receptors in skin [23] or mast cells [24].

**Figure 3:**
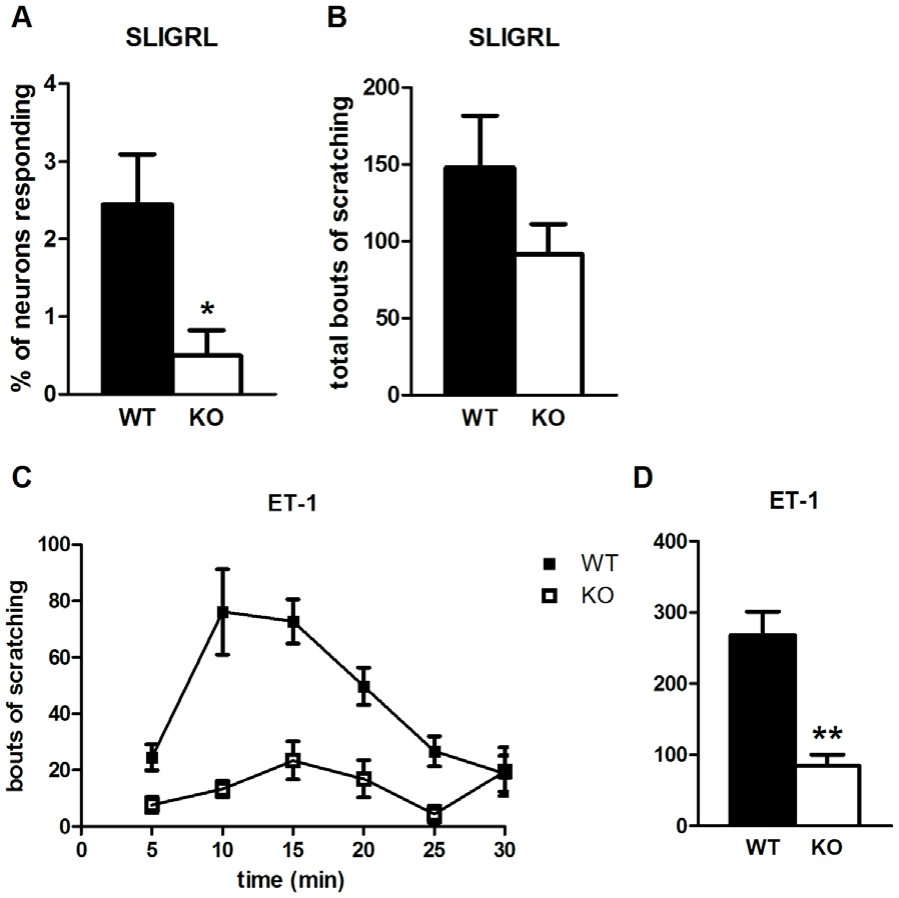
SLIGRL- and ET-1-induced responses are Pirt-dependent. (**A**) Cellular responses to SLIGRL (130 µM) are fewer in DRG neurons from *Pirt^−/−^* mice (n = 5 per genotype). (**B**) Pirt mutants (n = 11) scratch less than WT mice (n = 13) upon SLIGRL injection (0.1 µmol), but the difference is not statistically significant. (**C**, **D**) ET-1 (10 pmol) generates a decreased behavioral response in Pirt KO (n = 10) versus WT (n = 9). * *p*<0.05, ** *p*<0.001; two-tailed unpaired *t*-test.

The peptide Ser-Leu-Ile-Gly-Arg-Leu-NH_2_ (SLIGRL), derived from the sequence of protease-activated receptor 2 (PAR2), which exhibits self-activation [25], produces a nonhistaminergic form of itch [26]. *In vitro*, we saw a marked reduction in the number of *Pirt^−/−^* DRG neurons responsive to SLIGRL (2.4% of WT neurons vs. 0.5% in KO, Fig. 3A). We observed a decrease in the number of scratching bouts upon SLIGRL injection (148 bouts in WT vs. 92 in KO, Fig. 3B), but this difference was not statistically significant (*p* = 0.16). This may be due to the more variable scratching response to SLIGRL in comparison to other pruritogens.

One of the most potent itch mediators is endothelin-1 (ET-1). This peptide is perhaps best known for its vasodilator activity [27], but it has also been shown to produce itch in human subjects [28], [29]. ET-1 induces a robust behavioral itch response in mice through interaction with the endothelin A receptor [30], [31]. We found that Pirt KO mice (85 bouts) showed greatly reduced scratching in comparison with WT mice (269 bouts, Fig. 3C, D). We did not observe DRG neuron activation by ET-1 in our *in vitro* preparation, suggesting ET-1 may mediate itch indirectly, e.g. through action at mast cells [32], [33]. Alternatively, there may be a neuronal response we are unable to detect by Ca^2+^ imaging as other work suggests ET-1 may directly activate peripheral fibers in rats and human subjects [34], [35].

Thus, beyond a critical role for Pirt in histaminergic itch, deficits in the response to several histamine-independent pruritogens tested, i.e. CQ, α-Me-5HT, and ET-1, demonstrate that Pirt is involved in nonhistaminergic itch as well. Importantly, these results go beyond our initial hypothesis of Pirt regulating TRPV1-dependent itch as CQ [7], [36], 5HT/α-Me-5HT [6], [7], and ET-1 [7] all induce behavioral itch responses that do not require TRPV1. This establishes a definite role for Pirt in itch sensation that is separate from, and potentially in addition to, its modulation of TRPV1 function. While Pirt was identified for its regulation of TRPV1 activity and related effects on temperature and pain sensation [3], these results hint at a broader mechanism of action such as interactions with other ion channels or plasma membrane components (see Discussion).

### Pirt contributes to mast cell-dependent itch

Besides itch that entails direct activation of peripheral sensory neurons by pruritogens, itch-inducing compounds can also operate indirectly. Activity through secondary cell types such as keratinocytes or mast cells [37] can lead to release of substances that act directly upon DRG neurons to produce itch sensation. These indirect effects can be in addition to peripheral activation, e.g. CQ appears to generate itch via activation of MrgprA3^+^ DRG neurons as well as degranulation of mast cells [19].

**Figure 4:**
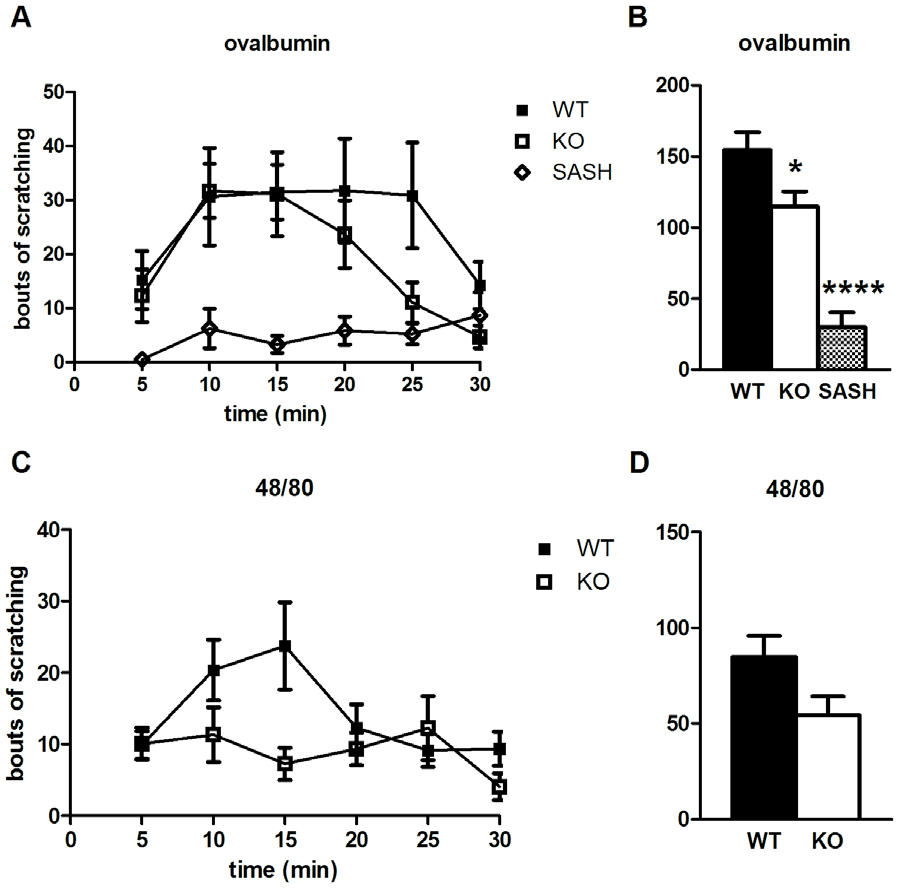
Pirt plays a role in mast cell-dependent itch. (**A**, **B**) In an allergy model, ovalbumin (50 µg) induces less scratching in *Pirt^−/−^* (n = 11) and SASH (n = 7) mice compared to WT controls (n = 8). (**C**, **D**) Pirt KO mice (n = 11) show a trend toward decreased scratching compared to WT (n = 13) when injected with compound 48/80 (30 µg), but this difference just misses statistical significance (*p*<0.06). * *p*<0.05, **** *p*<0.00001; two-tailed unpaired *t*-test.

One allergy model of itch makes use of the egg protein ovalbumin, an otherwise innocuous molecule that induces an immune response when co-injected with an adjuvant; subsequent injection of ovalbumin alone causes scratching [38]. We tested Pirt KO mice in this allergy model and found they exhibit significantly less scratching compared to WT (154 bouts vs. 115 bouts in KO, Fig. 4A, B). As mast cells are thought to play a critical role in the allergy response, we also tested SASH mice, which lack mast cells due to a chromosomal inversion affecting c-*kit* gene function [39]. They show drastically reduced scratching (30 bouts) in response to ovalbumin injection, verifying the role of mast cells in allergy-associated itch.

Another way to test mast cell-dependent itch is by inducing degranulation to release pruritogens. Compound 48/80 is an established mast cell degranulator that has been shown to produce itch in mice upon intradermal injection [9], [40]. *Pirt^−/−^* mice show a decreased scratching response to compound 48/80 (85 bouts in WT vs. 55 in KO, Fig. 4C, D), although this difference just misses statistical significance (*p*<0.06).

### Pirt displays specificity for different categories of itch

**Figure 5:**
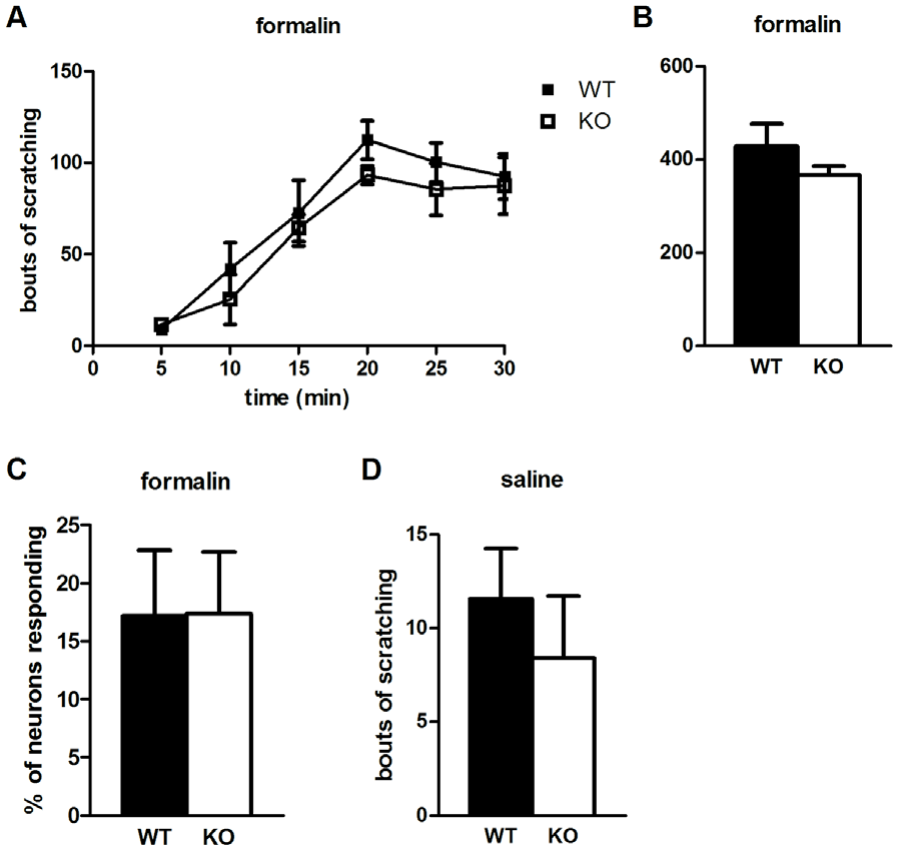
Pirt is not involved in all types of itch. (**A**, **B**) Pirt WT (n = 10) and KO (n = 8) mice display comparable formalin-induced (5%) itch behavior. (**C**) Ca^2+^ responses to formalin (0.02%) are unaltered in Pirt mutant DRG neurons (n = 6 per genotype). (**D**) Saline vehicle induces a similar baseline, i.e. spontaneous, scratching response in *Pirt^−/−^* (n = 5) and WT (n = 7) mice.

These results lead to the question of how broadly Pirt functions in itch and whether it is involved in all types of pruritoception. We tested another compound, formalin, which has long been used to model pain responses [41] although recent evidence suggests its acute effect may be pruritogenic [42], [43]. Interestingly, Pirt mice show scratching responses that do not differ significantly from WT controls (429 bouts in WT vs. 367 in KO, Fig. 5A, B). Formalin is known to directly activate the TRPA1 channel, which is also required for the compound's pain behavior [44], [45]. Earlier work suggests Pirt is not involved in TRPA1-dependent physiological or behavioral, i.e. formalin-induced pain, responses [3]. Consistent with these findings, we found *Pirt^−/−^* DRG neurons display Ca^2+^ responses to formalin that are indistinguishable from WT neurons (17.2% of WT neurons vs. 17.4% of KO, Fig. 5C), a response that is known to require TRPA1 [45]. This shows Pirt is not compulsory for all varieties of itch and reveals a degree of specificity in its function.

We also did not detect apparent changes in Pirt mice with respect to spontaneous itch behavior. Injection of saline vehicle produced minimal scratching in either WT or mutant mice (Fig. 5D). This suggests Pirt contributes to evoked scratch responses without affecting spontaneous itch.

## Discussion

We used behavioral and imaging methods to study the role of Pirt, a recently identified regulator of TRPV1 [3], in itch sensation. Large reductions in the response to histamine and HTMT in mutant mice show a clear role for Pirt in histaminergic itch. Nonhistaminergic itch as induced by CQ, α-Me-5HT, and ET-1 is also markedly decreased. Unexpectedly, although we observed a cellular change in the response to SLIGRL, the behavioral deficits in the KO were not significant. This may be explained by the high variability of behavioral responses obtained with this pruritogen.

Notably, these results allude to a mechanism that is independent of TRPV1, which does not participate in itch mediated by several compounds that produce scratching deficits in Pirt KO mice. This opens up a number of possibilities as to how Pirt functions mechanistically in itch sensation. Other TRP channels such as TRPV3 and TRPV4 have been implicated in itch [46]–[48] and Pirt may interact with these as it does with TRPV1. Beyond the TRP family, other ion channels found in DRG neurons may contribute to itch sensation, and furthermore, PIP_2_ has been linked to ion channel function in many contexts [49]. This provides a possible explanation if Pirt does not directly modify channel activity (see below). PIP_2_ and phosphoinositide signaling as a whole are also involved in other means of ion channel regulation such as membrane targeting and vesicle trafficking [49]. A recent study [50] suggests direct binding of TRPV1 to PIP_2_ may not be Pirt-dependent. However, the lack of a Pirt effect on TRPV1 is likely due to the concentration of capsaicin used in the study, i.e. 0.8–1.0 µM. We found a positive effect of Pirt only when a higher capsaicin concentration, i.e. 5 µM, was used to activate TRPV1 [3]. Moreover, as the deficits in *Pirt^−/−^* mice are not limited to TRPV1-dependent forms of itch, we do not expect PIP_2_ modulation of TRPV1 by Pirt to completely account for the various *Pirt^−/−^* phenotypes.

Considering many compounds are known to exert their pruritogenic effects by acting as ligands for GPCRs, including endothelin receptors, Mrgprs, and the histamine H1 receptor, Pirt may modulate some aspect(s) of the GPCR signaling cascade. The extensive network of the GPCR pathway presents various opportunities for regulation. For example, the enzyme phospholipase C β3 (PLCβ3) has been shown to be critical for several varieties of itch and likely acts downstream of particular GPCRs that serve as itch receptors [7], [11]. Thus, one possible mechanism is that Pirt modulates hydrolysis of PIP_2_ by PLCβ3. A recent study [51] has shown that chronic changes in PIP_2_ levels affect TRPV1 and TRPA1 activity in a concentration- and cell type-dependent manner, suggesting other sensory neuron factors like Pirt are important for PIP_2_-mediated TRP regulation. Specific G proteins, including both α and βγ subunits, may also have distinct roles in itch transduction [36], [52].

Another study [36] involving our laboratory found that TRPA1 is required for itch induced by CQ but not histamine. While seemingly contradictory, these results do not preclude roles for both Pirt and TRPA1 in detecting CQ itch. Pirt does not contribute to direct activation of TRPA1 by mustard oil or formalin, but because CQ generates itch by first directly activating MrgprA3 followed by downstream TRPA1 activation, the overall process can accommodate functions for both Pirt and TRPA1. Although it does not directly modify TRPA1 activity, Pirt may modulate PIP_2_ and/or other aspects of the G protein signaling pathway that constitutes CQ itch transduction.

*Pirt^−/−^* neurons show diminished Ca^2+^ responses to several compounds we tested. While these results are consistent with impaired signaling of these pruritogens, it remains to be demonstrated whether the deficits are directly linked to itch. For example, histamine elicits responses in a relatively broad population in DRG that is probably not limited to the neurons required for detecting itch. Work from our laboratory and others [1], [2] has indicated the existence of itch-sensing neurons in the DRG that are a subset of nociceptive neurons and would produce itch sensation upon activation. It would be worthwhile to examine if the reduction in histamine responsiveness found in *Pirt^−/−^* neurons is restricted to itch-sensing neurons, e.g. the MrgprA3-expressing population described recently [19], [36]. This can also be addressed by asking whether the targeted removal of Pirt from these purported itch-sensing DRG neurons produces cellular and behavioral deficits akin to those in the Pirt global KO mouse.

Our results provide further evidence that multiple molecular (TRPV1-dependent and -independent) and cellular (direct and indirect activation of sensory fibers) pathways are involved in itch signaling and demonstrate that Pirt plays a role in many of them. We have shown the presence of Pirt is indispensable for proper itch sensation. How it fits into this process mechanistically remains to be determined and may elucidate the different signaling pathways required to produce all varieties of itch. As Pirt is found in over 80% of DRG neurons [3], it is probable that it serves functions beyond thermal pain and now itch. Its mechanism of action in these contexts may shed light on other somatosensory processes to which Pirt contributes.

At the same time, the specific expression pattern of Pirt, which is limited to sensory neurons, indicates its potential use as a target for itch therapeutics. There are a number of mouse models to investigate various aspects of medical itch [53]–[58] that can be utilized to see if Pirt plays a role in any facet of itch in its various pathological manifestations such as inflammatory, dermatitis, etc. Our results establish a critical role for Pirt in sensing itch and generate new questions to advance knowledge of itch from both basic science and clinical perspectives.

## Materials and Methods

### *Pirt^−/−^* mice

The mice were generated as previously described [3]. Briefly, the entire *Pirt* coding region was replaced with an EGFPf-IRES-rtTA-ACN targeting construct to produce a null allele. Heterozygotes were mated to produce *Pirt^−/−^* mice (KO) and *Pirt^+/+^* littermates (WT).

### DRG neuron cell culture

DRGs were dissected from all spinal levels of 3–8 week old mice. These were collected in ice cold DH10 medium (90% DMEM/F-12, 10% FBS, 100 U/ml penicillin, 100 µg/ml streptomycin, Gibco) and digested at 37°C in enzyme solution (5 mg/ml dispase, 1 mg/ml collagenase type I in HPBS without Ca^2+^ or Mg^2+^, Gibco). After trituration and centrifugation, cells were resuspended in DH10 medium supplemented with NGF (20 ng/ml) and GDNF (25 ng/ml) and plated on glass coverslips coated with poly-D-lysine (0.5 mg/ml) and laminin (10 µg/ml). These were cultured in an incubator (95% O_2_ and 5% CO_2_) and used for Ca^2+^ imaging after 18–24 h.

### Ca^2+^ imaging

Neurons were loaded with fura-2-acetoxymethyl ester (Molecular Probes) for 30 min in the dark at room temperature. Cells were washed and imaged using 340 and 380 nm excitation to detect intracellular free Ca^2+^. Neurons were tested for responses to each compound in at least four independent experiments with a minimum of 100 cells analyzed each time.

### Itch behavior assay

Mice tested were 2- to 3-month old males that had been backcrossed to C57Bl/6 mice for at least ten generations. Pruritogens were dissolved in a 50 µl volume of saline and subcutaneously injected into the nape of the neck. Bouts of scratching behavior were counted for 30 min immediately afterward with one bout defined as continuous scratching movements by the hindpaws directed at the area near the injection site.

The allergy model [38] was performed as follows: 50 µg ovalbumin dissolved in phosphate-buffered saline, with 2 mg aluminum hydroxide gel, was administered intraperitoneally, twice at two week intervals. Two weeks after the second sensitization, 50 µg ovalbumin dissolved in saline was administered in the same manner as other pruritogens and scratching behavior was quantified. All behavioral tests were performed by an experimenter blind to genotype and were done under the protocol approved by the Animal Care and Use Committee of Johns Hopkins University School of Medicine (Protocol number: MO09M354; Approval date: 07/22/2010).

### Data analysis

Data are presented as mean ± standard error of the mean (SEM). Statistical comparisons were performed using an unpaired Student's *t*-test with equal or unequal variances based on F-test results. Differences were considered statistically significant at *p*<0.05.
